# Editorial: Epigenetics of Neurodevelopmental, Neuromuscular and Neurodegenerative Disorders

**DOI:** 10.3389/fnmol.2022.948827

**Published:** 2022-06-10

**Authors:** Fabio Coppedè, Cristina Cereda, Carla Lintas, Andrea Stoccoro

**Affiliations:** ^1^Department of Translational Research and of New Surgical and Medical Technologies, University of Pisa, Pisa, Italy; ^2^Department of Women, Mothers and Neonatal Care, Children's Hospital V. Buzzi, Milan, Italy; ^3^Laboratory of Medical Genetics, University Campus Bio-Medico of Rome, Rome, Italy

**Keywords:** epigenetics, DNA methylation, miRNA, neurodevelopmental disorders, autism, amyotrophic lateral sclerosis, neurodegenerative diseases

Epigenetic mechanisms are fundamental for the development and function of the central nervous system, and their impairment contributes to neurodevelopmental (Reichard and Zimmer-Bensch, 2021), neuromuscular (Coppedè, 2020), and neurodegenerative disorders (Gagliardi et al., 2018; Bellver-Sanchis et al., 2021). As such, active research is ongoing to identify epigenetic biomarkers of these disorders, and both DNA methylation changes and circulating RNAs are increasingly recognized as potential biomarkers to improve diagnosis and to monitor disease status and response to therapeutic interventions (Sproviero et al., 2021; Hirunagi et al., 2022; Kaur et al., 2022). Compounds targeting histone tail modifications, as well as RNA interfering techniques and genome editing approaches, are currently explored as potential therapeutic tools in these disorders (Coppedè, 2022). In addition, active research is devoted to the identification of genetic and environmental factors, as well as early-life events, contributing to impaired epigenetic regulation of gene expression in neurons (Miguel et al., 2019).

With this special issue, we aim to showcase recent advances in epigenetics of neurodevelopmental, neuromuscular, and neurodegenerative disorders. We present seven articles covering themes of early-life exposure, neurodevelopmental disorders resulting from mutations of genes of the epigenetic machinery, identification of epigenetic disease biomarkers, epigenetic properties of drugs, and gene-environment interactions.

Concerning neurodevelopmental disorders, Alberry et al. reviewed the epigenetic consequences of prenatal ethanol exposure followed by exposure to early life stress in the postnatal period, such as abuse or neglect during early development, in the context of fetal alcohol spectrum disorders (FASDs), a heterogeneous group of neurodevelopmental disorders that begin *in-utero*, manifest during childhood, and last a lifetime. Despite that FASDs result from maternal alcohol consumption during pregnancy, the authors discuss how a stressful postnatal environment may worsen their outcomes, emphasizing the role of dynamic epigenetic changes during the neurodevelopmental continuum, from prenatal to postnatal stages, in the development of FASDs. Authors observed that epigenetic and gene expression changes induced by both prenatal ethanol exposure and exposure to early-life stress in the postnatal period converge at the clustered protocadherin locus and oxidative stress pathways, suggesting that targeting these pathways may be a promising avenue for therapeutic strategies in the future. The interesting message from this article is the combined effect of pre- and postnatal exposures in shaping the neurodevelopmental epigenome, and evidence that postnatal adverse exposures can worsen the epigenetic consequences of *in-utero* exposure.

The identification of early-life epigenetic biomarkers of neurodevelopmental conditions is a timely and attractive issue, that is further addressed by the research article by Bakulski et al. In this manuscript authors present the results of a prospective study aimed at identifying early epigenetic signatures of autism spectrum disorders (ASDs). They performed a genome-wide DNA methylation analysis in cord blood, maternal blood in early and late pregnancy, maternal placenta, and fetal placenta to estimate associations between DNA methylation in these tissues and later diagnosis of ASD, observing that DNA methylation sites nominally associated with a later ASD diagnosis across the five investigated tissues were enriched for ASD risk genes from the Simons Foundation Autism Research Initiative (SFARI) database. Notably, this is the most comprehensive, multiple perinatal tissue investigation of epigenetics in ASD to date and suggests that, if replicated in subsequent studies, the identified methylation signatures could represent early biomarkers to identify individuals at risk to develop an ASD.

Interestingly, several neurodevelopmental disorders result from mutations in genes coding from proteins that add, remove, or read epigenetic marks, collectively referred to as genes of “the epigenetic machinery.” Recent investigations suggest that mutations in these genes can result in global dysregulation of epigenetic patterns, leading to epigenetic signatures that are increasingly gaining interest as diagnostic tools for neurodevelopmental disorders (Aref-Eshghi et al., 2021; Coppedè, 2021). Within this context, the review article by Wang et al. provides an overview of pre-clinical studies on the role of SETD1A, a chromatin remodeler that influences gene expression through the modulation of mono- di- and trimethylation marks on Histone-H3-Lysine-4 (H3K4me1/2/3), in neuronal development. Furthermore, authors review recent evidence of *SETD1A* mutations linked to neurodevelopmental and neuropsychiatric disorders.

Two articles in this special issue deal with non-coding RNAs as disease biomarkers. Individuals with neurodevelopmental disorders are often at higher risk for depressive disorders, such as major depressive disorder (MDD) (Watanabe, 2021). Among micro-RNAs (miRNAs), a dysregulated miR-124 expression has been often observed in animal models of MDD, as well as in human brain and blood tissues of MDD patients (Dwivedi, 2017). The research article by Zeng et al. was aimed to further explore the mechanisms leading to altered miR-124 expression in MDD, and authors observed that MDD patients display hypomethylation of the miR-124 precursor genes compared to healthy matched controls, likely representing a biomarker for MDD.

Amyotrophic lateral sclerosis (ALS) is one of the major and fatal neurodegenerative disorders, resulting from motor neuron degeneration. Epigenetic dysregulation in ALS has been reported at the level of DNA methylation, histone tail modifications and expression of circulating non-coding RNAs and miRNA in plasmatic extracellular vesicles (Gagliardi et al., 2018; Coppedè, 2020). In this special issue, the article by Lo et al. provides evidence that extracellular vesicles in serum and central nervous tissues from ALS patients contain overlapping dysregulated miRNAs associated with common biological pathways altered in neurodegeneration, including axon guidance and long-term potentiation, providing further insights into the pathological mechanisms of the disease and potential circulating biomarkers.

Dai et al. investigated the epigenetic properties of lovastatin, a lipid-lowering drug, in cell models of Parkinson's disease. They showed that lovastatin alleviates α-synuclein aggregation and phosphorylation in cellular models of synucleinopathy, reducing oxidative stress, modulating histone tail modifications, and inhibiting the expression of a protein kinase involved in α-synuclein phosphorylation.

In recent years, genome-wide association studies provided a powerful tool to detect many common genetic variants associated with human traits and diseases, variants that can be combined to create polygenic risk scores (PRSs) for these conditions. Therefore, recent strategies to investigate gene-environment interactions in complex disorders include the possibility to test the contribution of environmental factors in individuals stratified according to their PRS, i.e., in individuals at high or low genetic risk for a complex disorder. In their research article, Liu et al. provide evidence that healthy lifestyles, including never smoking, moderate drinking and active physical activity, lower the risk of cognitive impairment in older adults at high PRS for obesity, and discuss the potential epigenetic effects of these lifestyles.

In summary, the seven articles presented in this collection cover a breadth of topics converging on epigenetic mechanisms related to neuronal activity and degeneration. We believe this Research Topic will be of interest to researchers working on areas including neurodevelopment, neurobehavior and neurodegeneration.

## Author Contributions

FC drafted the manuscript. All authors contributed to the completion of the work and approved its submission.

## Conflict of Interest

The authors declare that the research was conducted in the absence of any commercial or financial relationships that could be construed as a potential conflict of interest.

## Publisher's Note

All claims expressed in this article are solely those of the authors and do not necessarily represent those of their affiliated organizations, or those of the publisher, the editors and the reviewers. Any product that may be evaluated in this article, or claim that may be made by its manufacturer, is not guaranteed or endorsed by the publisher.
